# Triglyceride-glucose index predicts the mortality risk among incident peritoneal dialysis patients in a cohort study

**DOI:** 10.1038/s41598-025-19171-8

**Published:** 2025-10-09

**Authors:** Kao-Ming Hsu, Yao-Peng Hsieh, Yu-Jun Chang, Shr-Mei Tsai, Ping-Fang Chiu

**Affiliations:** 1 Division of Nephrology, Department of Internal Medicine, Changhua Christian Hospital, 135 Nanxiao Street, Changhua, 500 Taiwan, ROC; 2 Department of Post Baccalaureate Medicine, College of Medicine, National Chung Hsing University, Taichung, Taiwan; 3 Big Data Center, Changhua Christian Hospital, Changhua, 50006 Taiwan; 4 Department of Nursing, Changhua Christian Hospital, Changhua, Taiwan

**Keywords:** Cardiovascular disease, Chronic kidney disease, Mortality, Peritoneal dialysis (PD), Triglyceride-glucose (TyG) index, Diseases, Health care, Medical research

## Abstract

Insulin resistance (IR) frequently cause higher levels of fasting glucose and triglyceride. Triglyceride- glucose (TyG) index has been suggested as a simple, reliable, and cost-effective surrogate for IR. We conducted the present study to assess whether TyG index is associated with increased risk of all-cause and cardiovascular (CV) mortality among our PD cohort. This observational study included 553 patients initiating PD between 2003 and 2017 who were divided into three groups by TyG tertiles. The study exposure was the TyG index at study enrollment. Associations of TyG index with patient mortality were examined in Cox models and the potential confounding covariates included medication use, demographic, comorbidities, PD-associated and laboratory data. The optimal cut-off points of TyG index were also determined using the receiver operating characteristic (ROC) analysis and area under ROC curve (AUC) was calculated. During follow-up, 142 patients died, of whom 89 CV deaths occurred. The risks of all-cause and CV mortality increased with tertiles of TyG index. In the multivariable-adjusted models, the hazard ratios (HRs) in tertile 3 versus tertile 1 were 2.12 (95% CI 1.31–3.43, *p* = 0.021) and 2.78 (95% CI 1.34–5.76, *p* = 0.006) for all-cause and CV mortality, respectively. Those independent associations remained even when TyG index was treated as a continuous variable, or per 1-standard deviation increase. The cut-off point of TyG index was 8.79 (62.7% sensitivity and 61.6% specificity) with AUC of 0.652 and 8.85 (66.3% sensitivity and 62.9% specificity) with AUC of 0.681 for all-cause and CV mortality, respectively. Elevated levels of TyG index significantly predicted increased risks of all-cause and CV mortality in patients initiating PD. More studies are required to compare with other surrogates of insulin sensitivity and extrapolated to other ethnic populations.

## Introduction

Because of prolonged life span and accompanying comorbidities burden, chronic kidney disease (CKD) has been increasingly prevalent and attracted global health concern due to its impact on medical and socioeconomic costs^1^. CKD has been recognized as a cardiovascular (CV) risk equivalent and it, particularly end-stage renal disease (ESRD), leads to premature death by a variety of traditional and untraditional risk factors, such as mineral disorders, oxidative stress and inflammation^2,3^. Peritoneal dialysis (PD) as one of renal replacement treatment is more flexible and tailored than intermittent hemodialysis for ESRD patients. From 2009 to 2019, the percentage of incident dialysis patients performing PD increased from 6.6 to 12.3% in the United States^4^. In spite of improvement in patient education, dialysate exchange technique and personalized precision medicine, the mortality rate of PD patients remains high^5^.

Besides its pivotal role in the pathogenesis of diabetes mellitus (DM), insulin resistance (IR) recently has been extensively shown to be associated with not only hypertension, dyslipidemia and obesity, but also an independent risk for the progression of CKD and cardiovascular disease, which is the leading cause of death in CKD patients^6–11^. Previous studies even reported the presence of IR through the impaired glucose metabolism and insulin homeostasis in early stage of CKD via various mechanisms^12^. These metabolic alterations may be augmented in PD patients because of the exposure to high glucose load in the dialysate, resulting poor patient survival.

The hyperinsulinemia- euglycemic clamp testing is the gold-standard method for the assessment of IR and the homeostasis model assessment of IR (HOMA-IR) is commonly used by measuring circulating fasting insulin and glucose concentrations^13^. However, these testing practices are either complexing, costly or unavailable in routine clinical settings or large-scale studies. Instead, triglyceride- glucose (TyG) index has been suggested as a simple, reliable, and cost-effective surrogate for IR, assessed by HOMA-IR and hyperinsulinemia- euglycemic clamp, in many recent studies^14,15^. Few studies have evaluated the association of TyG index with mortality in PD patients. Thus, we carried out the present study to assess the value of TyG in predicting mortality risk in our PD cohort.

## Materials and methods

### Participants and measurements

This was a retrospective cohort study conducted in a single PD unit of a middle Taiwan medical center. Data were retrieved and analyzed from reviewing well-established medical records for patients who underwent PD for ESRD in Changhua Christian Hospital. Our study cohort consisted of patients who were over 18 years old, initiated PD from 2003 to 2017 and survived on PD for more than 3 months. The study outcomes of interest consisted of all-cause and CV mortality. All the research procedures were approved by the Ethics committee of Changhua Christian Hospital and carried out in line with the declaration of Helsinki. Informed consents for each participant were waived when a retrospective survey was conducted using an anonymized dataset in Taiwan.

### Data collection

We collected various clinical, demographic, anthropometric, laboratory and PD data at baseline for adjustments. Demographics included age, gender, and body mass index (BMI) which was defined as weight (kg) divided by squared height (m^2^). Clinical comorbid conditions were recorded at study entry and consisted of diabetes mellitus (DM), hypertension and cardiovascular disease. PD data included peritoneal equilibrium tests and dialysis adequacy evaluation. Total weekly urea clearance (Kt/V), normalized protein catabolic rate (nPCR), and dialysate-to-plasma creatinine ratio (D/P creatinine) at 4 h were calculated. Residual kidney function was determined from the mean of 24-hour renal clearance of urea and creatinine. Blood samples were drawn and tested for serum creatinine, blood urea nitrogen (BUN), albumin, glutamic-pyruvic transaminase (GPT), white blood cell (WBC) count, hemoglobin, ferritin, fasting blood glucose (FBG), glycated hemoglobin (HbA1C), total cholesterol (TC), triglyceride (TG), low-density lipoprotein cholesterol (LDL-C), and high-density lipoprotein cholesterol (HDL-C), intact parathyroid hormone (PTH), uric acid, calcium, and phosphate. Pharmacotherapy of angiotensin-converting enzyme (ACE) inhibitors or angiotensin II receptor blockers (ARB) and lipid-lowering drugs was also recorded.

### Exposure definition

TyG index was calculated as the ln (fasting triglyceride [mg/dL] × fasting glucose [mg/dL]/2)^16^. Study cohort was classified into 3 groups by tertiles of TyG index: tertile 1 (TyG index ≦ 8.40), tertile 2 (8.40 < TyG index < 8.98) and tertile 3 (TyG index ≥ 8.98).

### Statistical analysis

As the TyG index is a continuous metabolic marker with no universally accepted clinical cut-off, to enhance interpretability and comparability, we categorized it into tertiles based on its distribution in our study population. This data-driven approach is commonly used in epidemiological studies when established thresholds are lacking, as it allows for a balanced distribution of participants and facilitates comparisons across exposure levels. Several prior studies investigating similar metabolic indices or risk scores have also adopted tertile or quartile stratifications for exploratory or comparative purposes^17,18^.

The TyG index was used to divide the whole study cohort into three groups (tertiles) to investigate the associations of TyG index with clinical outcomes. Descriptive baseline patient characteristics were shown as frequencies and percentages for categorical variables, and mean ± standard deviation (SD) or median and interquartile range (IQR) for continuous variables with normal or skewed distribution, respectively. The differences across the TyG tertiles were compared using the analysis of variance (ANOVA) or the Kruskal-Wallis test for continuous variables, while the categorical variables were compared by Chi-square test or Fisher’s exact test.

Comparisons of CV and all-cause mortality were performed by plotting Kaplan-Meier survival curve and the log-rank tests determined the survival differences among the three groups. We conducted tests using Schoenfeld residuals (cox.zph in R) to evaluate the proportional hazards (PH) assumption in our Cox regression models. For all-cause mortality, the test result for the main exposure variable TyG showed a non-significant p-value (Chi-square = 0.03, df = 1, *p* = 0.87), suggesting that the PH assumption was not violated. Similarly, for cardiovascular mortality, the PH test result for TyG was also non-significant (Chi-square = 1.58, df = 1, *p* = 0.21). These results support the use of the Cox proportional hazards model in our analysis. Multivariate-adjusted Cox proportional hazard models was estimated to assess independent associations of TyG index with study outcomes after accounting for those baseline variables contributing significantly.

Five models were used for stepwise adjustments for clinical confounding factors: Model 1 was adjusted for sex, age, smoking status and BMI; Model 2 was further adjusted for medications; Model 3 was further adjusted for comorbidities; Model 4 was further adjusted for PD-related parameters; Model 5 was further adjusted for laboratory data. The results of Cox regressions were showed as hazard ratios (HRs) and 95% confidence intervals (CIs).

We used G*Power software (version 3.1.9.2, Franz Faul, Universitat Kiel, Germany) to calculate the sample size required for the binary logistic regression analysis. Assuming that the odds ratio of death for each unit increase in Z-TyG index (Mean = 1, SD = 0) is 1.8, a Type I error rate of 0.05, a power of 0.8 and a mortality rate of 25% among dialysis patients, the minimum sample size required is 134.

In addition, to address the concern regarding the absence of conventional cut-offs, we used both a per 1-unit increase and a per 1-standard deviation (SD) increase in TyG in sensitivity analyses. The 1-unit increase reflects the raw scale of the index and allows direct assessment of the incremental risk. The 1-SD increase, on the other hand, standardizes the effect size and allows comparison with other biomarkers, particularly when there is no consensus on clinical thresholds. It is also widely used in epidemiologic studies to enable comparability across variables with different units or scales^19^.

There is no normal reference range for the TyG index, nor is there an optimal cut-off value. We conducted the receiver operating characteristic (ROC) curve analysis to determine the optimal cut-off point for TyG index and calculate the area under the ROC curve (AUC). All statistical analyses were performed using IBM SPSS Statistics for Windows, version 22.0 (IBM Corp., Armonk, NY) and R version 4.1.2. Two-sided p-values ≤ 0.05 were considered statistically significant.

## Results

### Patients’ baseline characteristics

A total of 553 ESRD patients on PD were eligible for this investigation from 2003 to 2017 in the current study. Baseline patient characteristics by tertiles of the TyG index were presented in Table 1. The mean age was 52.5 ± 15.2 years and 265 (47.9%) were men. Patients with a higher TyG index were more likely to be older, have higher prevalence of diabetes and CVD, lower levels of BUN, albumin, HDL-C, creatinine, phosphate, and iPTH, higher levels of calcium, LDL-C, ferritin and WBC counts compared with TyG index tertile 1 group.

**Table 1:** Baseline characteristics of the study population by the TyG groups.

	TyG index tertile 1	TyG index tertile 2	TyG index tertile 3	*p*-Value
	(TyG ≤ 8.40)	(8.40 < TyG < 8.98)	(TyG ≥ 8.98)	
Number of patients	187	182	184	--
Sex, men	102 (54.5%)	81 (44.5%)	82 (44.6%)	0.083
Age (years)	49.2 ± 15.7	52.1 ± 15.6	56.3 ± 13.4	< 0.001
Body mass index (kg/m^2^)	23.2 ± 4.1	24.6 ± 4.0	23.5 ± 3.9	< 0.001
Smoking				
Never	148 (79.1%)	159 (87.7%)	149 (81%)	0.143
Current	4 (2.1%)	2 (1.1%)	7 (3.8%)	
Ever	35 (18.7%)	21 (11.5%)	28 (15.2%)	
Comorbidity disease				
Hypertension	169 (90.4%)	154 (84.6%)	159 (86.4%)	0.238
Diabetes mellitus	40 (21.4%)	41 (22.5%)	109 (59.2%)	< 0.001
Cardiovascular disease	51 (27.3%)	60 (33%)	80 (43.5%)	0.004
Medication use				
ACEI/ARB	122 (65.2%)	109 (59.9%)	122 (66.3%)	0.392
Lipid-lowering agents	30 (16%)	38 (20.9%)	62 (33.7%)	< 0.001
Laboratory data				
Albumin (g/dL)	3.4 (3.0–3.8)	3.3 (3.0–3.8)	3.2 (2.9–3.7)	0.012
Fasting glucose (mg/dL)	93.0 (84.0-100.0)	97.5 (91.0-106.0)	133.5 (100.5–186.0)	< 0.001
Blood urea nitrogen (mg/dL)	87 (76–102)	82 (70–98)	79.5 (67.5–97)	0.003
Creatinine (mg/dL)	10.1 (8.6–12.6)	9.7 (8.2–12.1)	9.1 (7.7–10.7)	< 0.001
Calcium (mg/dL)	8.2 (7.7–8.7)	8.3 (7.9–8.9)	8.4 (8.1–8.9)	0.004
Phosphate (mg/dL)	5.6 (4.8–6.8)	5.6 (4.9–6.4)	5.3 (4.5–6.2)	0.014
GPT (U/L)	16.0 (12.0–23.0)	17.0 (12.0–24.0)	16.0 (12.0–23.0)	0.891
Cholesterol (mg/dL)	166 (141–209)	189.5 (163–224)	211.5 (168.5–249)	< 0.001
HDL-C (mg/dL)	58(49–69)	50(39–62)	41 (33–52)	< 0.001
LDL-C (mg/dL)	99.6 (76-126.2)	115 (93-141.8)	115.8 (85.5-144.3)	0.001
Triglyceride (mg/dL)	80.5 (63–100)	119 (103–146)	167 (127–227)	< 0.001
Hemoglobin (g/dL)	8.6 (7.6–9.52)	8.7 (7.8–9.4)	8.9 (8.0–9.6)	0.139
Ferritin (ng/mL)	193.2 (112.6-372.2)	229.5 (118.8-385.2)	257.8(127.6-473.1)	0.034
HbA1C (%)	5.1 (4.8–5.6)	5.1 (4.8–5.4)	5.5 (5.1–6.3)	< 0.001
Uric Acid (mg/dL)	7.0 (6.1–7.9)	7.4 (6.3–8.4)	7.3 (6.5–8.3)	0.013
Intact PTH (pg/mL)	358.5(201.7–606)	345.5(193.7–597)	299 (181–473)	0.044
WBC (× 10^3^/µL)	6.5 (5.3–7.7)	6.5 (5.5–8.2)	7.6 (6.5–9.6)	< 0.001
PD-related parameters				
D/P creatinine at 4 h	0.7 (0.6–0.8)	0.7 (0.6–0.8)	0.7 (0.6–0.8)	0.096
Weekly total Kt/V urea	2.1 (1.8–2.4)	2.0 (1.7–2.3)	2.0 (1.7–2.2)	0.008
Daily nPNA (g/kg)	1.1 (0.9–1.3)	1.1 (0.8–1.2)	1.0 (0.8–1.2)	< 0.001
Residual renal function (mL/min/1.73 m2)	3.1 (2.3–4.1)	2.9 (2.1–3.6)	3.0 (1.9–4.4)	0.266

Values are expressed as mean ± SD, median (interquartile range) or number (percentage).

The differences across the TyG tertiles were compared using the analysis of variance (ANOVA) or the Kruskal-Wallis test for continuous variables, while the categorical variables were compared by Chi-square test or Fisher’s exact test.

ACE inhibitor, angiotensin-converting enzyme inhibitor; ARB, angiotensin II receptor blocker; BMI, body mass index; WBC, white blood cell count; PTH, parathyroid hormone; nPNA, normalized protein nitrogen appearance; D/P creatinine, dialysate-to-plasma creatinine ratio; HDL-C: high density lipoprotein- cholesterol; LDL-C: low density lipoprotein- cholesterol.

### Association of TyG index with all-cause mortality

**Fig. 1:**
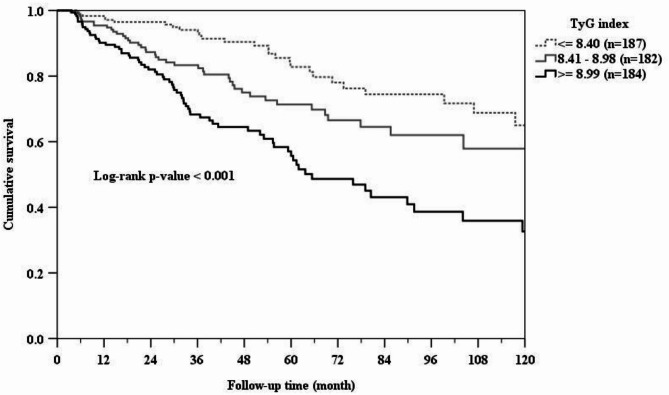
Kaplan-Meier curve of overall patient survival according to the TyG index groups (log-rank test, *p* < 0.001).

During a mean follow-up of 3.83 ± 3.12 years, 142 (25.7%) deaths occurred. The crude rate of all-cause mortality increased substantially with 30 deaths (16%) for TyG index tertile 1, 43 deaths (23.6%) for tertile 2 and 69 deaths (37.5%) for tertile 3 (*p* < 0.001). Kaplan- Meier survival curve also revealed significant differences (log-rank test p- value < 0.001) with patients in tertile 3 of the TyG index having a higher risk of all-cause mortality than patients in the other groups during the study period (Fig. 1). Table 2 listed the unadjusted and adjusted risks of all-cause mortality in reference to tertile 1 group of the TyG index. The crude HRs of all-cause mortality increased over time by TyG tertiles. Patients in the TyG tertile 2 was associated with an HR of 1.74 (95% CI, 1.09–2.78) and those in the TyG tertile 3 was associated with an HR of 3.06 (95% CI, 1.99–4.71) compared with TyG tertile 1. Moreover, compared with tertile 1, the fully adjusted HRs (model 5) were 1.61 (95% CI, 0.99–2.62), and 2.12 (95% CI, 1.31–3.43) for tertiles 2 and 3, respectively.

**Table 2:** Univariate and multivariate Cox regression models of all-cause and CVD mortality for TyG groups.

TyG category: TyG tertile 1 group: TyG index ≤ 8.40 (as reference group); TyG tertile 2 group: 8.40 < TyG index < 8.98 TyG tertile 3 group: TyG index ≥ 8.98		All-cause mortality		CVD mortality	
		Hazard ratio (95% CI)	*p*-value	Hazard ratio (95% CI)	*p*-value
(A) Crude model (versus tertile 1)	Tertile 2	1.74 (1.09–2.78)	0.02	2.58 (1.34–4.99)	0.005
	Tertile 3	3.06 (1.99–4.71)	< 0.001	4.82 (2.60–8.91)	< 0.001
Model 1 (versus tertile 1)	Tertile 2	1.64 (1.03–2.63)	0.039	2.49 (1.28–4.82)	0.007
	Tertile 3	2.71 (1.75–4.21)	< 0.001	4.34 (1.85–6.40)	< 0.001
Model 2 (versus tertile 1)	Tertile 2	1.44 (0.90–2.32)	0.127	2.14 (1.10–4.15)	0.025
	Tertile 3	2.25 (1.45–3.47)	< 0.001	3.44 (1.85–6.40)	< 0.001
Model 3 (versus tertile 1)	Tertile 2	1.60 (1.00-2.56)	0.049	2.27 (1.17–4.40)	0.015
	Tertile 3	2.10 (1.33–3.31)	0.001	2.65 (1.40–5.01)	0.003
Model 4 (versus tertile 1)	Tertile 2	1.23 (0.75–2.01)	0.413	2.27 (1.16–4.44)	0.017
	Tertile 3	1.61 (1.01–2.58)	0.046	2.32 (1.17–4.58)	0.016
Model 5 (versus tertile 1)	Tertile 2	1.61 (0.99–2.62)	0.053	2.16 (1.05–4.40)	0.035
	Tertile 3	2.12 (1.31–3.43)	0.021	2.78 (1.34–5.76)	0.006
(B) Sensitivity tests					
(i) TyG index per 1-unit increase in model 5	–	1.62 (1.26–2.08)	< 0.001	1.45 (1.03–2.04)	0.033
(ii) TyG index per 1- SD increase in model 5	–	1.39 (1.72–1.66)	<0.001	1.29 (1.02–1.63)	0.033

Crude model: crude HR of TyG category. Estimated hazard ratios were derived from Cox’s proportional hazard models.

Model 1: TyG index, age, sex, BMI, and smoking status.

Model 2: Model 1 plus medications (ACE inhibitors/ARB and lipid-lowering agents).

Model 3: Model 2 plus comorbidities (diabetes mellitus, hypertension and cardiovascular disease).

Model 4: Model 3 plus PD related parameters (weekly total Kt/V urea, nPNA, D/P creatinine at 4 h and residual renal function).

Model 5: Model 4 plus laboratory data (albumin, creatinine, blood urea nitrogen, WBC counts, ferritin, hemoglobin, cholesterol, LDL-C, HDL-C, uric acid, intact PTH, glutamic-pyruvic transaminase (GPT), calcium and phosphorus).

TyG index, triglyceride- glucose index; CVD, cardiovascular disease.

### Association of TyG index with CV mortality

**Fig. 2:**
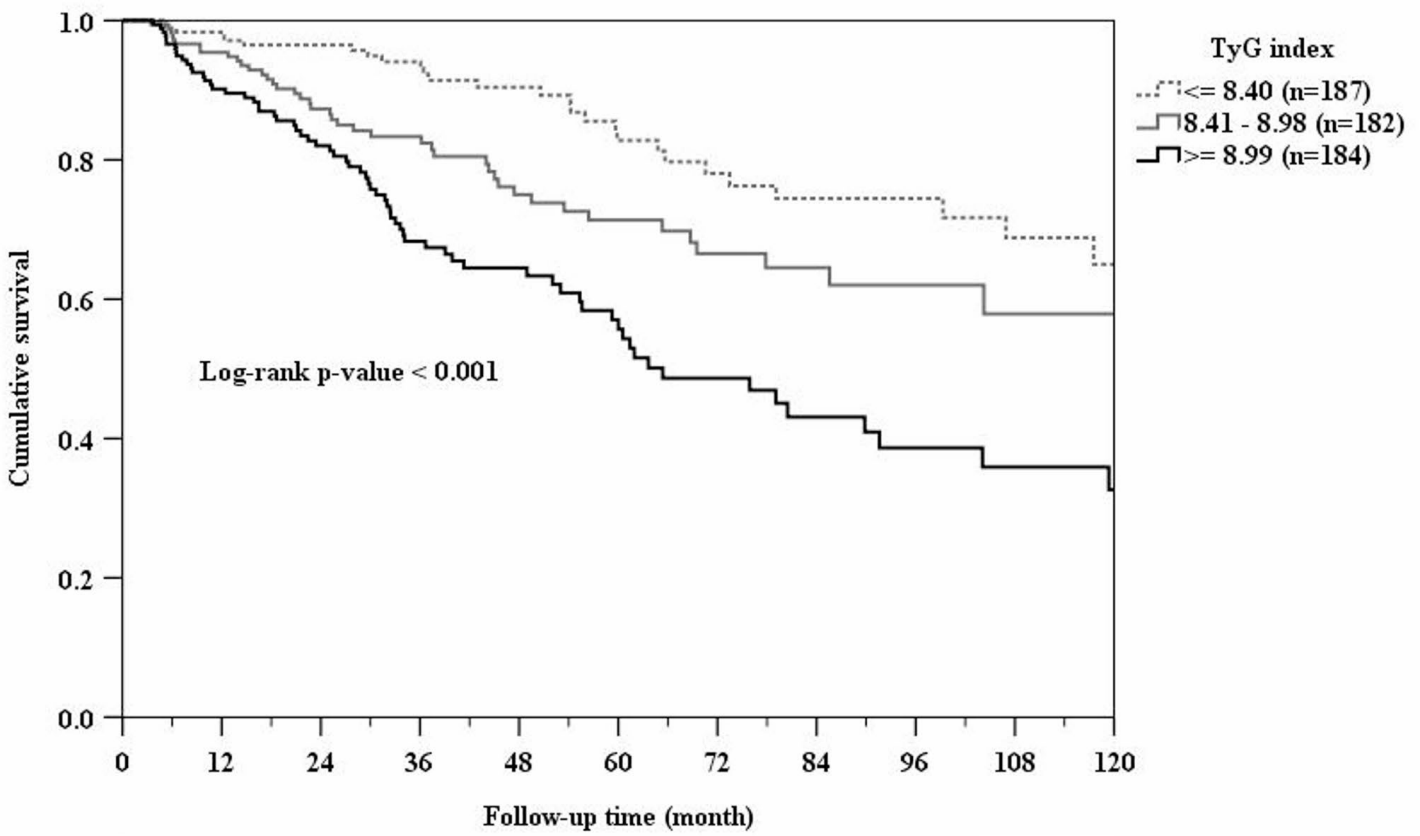
Kaplan-Meier curve of cardiovascular survival according to the TyG index groups (log-rank test, *p* < 0.001).

During the study period, 89 (16.1%) CV deaths occurred. The crude rate of CV mortality increased substantially with 13 CV deaths (7%) for TyG index tertile 1, 28 CV deaths (15.4%) for tertile 2 and 48 CV deaths (26.1%) for tertile 3 (*p* < 0.001). Kaplan- Meier survival curve also revealed significant differences (log-rank test p- value < 0.001) with patients in tertile 3 of the TyG index having a higher risk of CV mortality than patients in the other groups during the study period (Fig. 2). Table 2 listed the unadjusted and adjusted risks of CV mortality in reference to tertile 1 of the TyG index. The crude HRs of CV mortality increased over time by TyG tertiles. Patients in the TyG tertile 2 was associated with an HR of 2.58 (95% CI, 1.34–4.99) and those in the TyG tertile 3 was associated with an HR of 4.82 (95% CI, 2.60–8.91) compared with TyG tertile 1. Moreover, compared with tertile 1, the fully adjusted HRs (model 5) were 2.16 (95% CI, 1.05–4.40), and 2.78 (1.34–5.76) for tertiles 2 and 3, respectively.

### Sensitivity analysis

Two distinct analyses were performed to test the consistence of our findings in the sensitivity tests (Table 2). When the TyG index was treated as a continuous variable, the risks of all-cause and CV mortality were associated with adjusted HRs of 1.45 (1.03–2.04) and 1.62 (1.26–2.08) for every 1- unit increase of TyG index, respectively. Furthermore, a per 1-SD increase of the baseline TyG index was associated with 29% (HR, 1.29; 95% CI, 1.02–1.63) and 39% (HR, 1.29; 95% CI, 1.72–1.66) higher risks of all-cause and CV mortality, respectively.

### The optimal TyG index cut- off points and AUC

Among 553 PD patients in our study cohort, the optimal cut-off points of TyG index for all-cause and CV mortality were calculated to be 8.79 with 0.627 sensitivity and 0.616 specificity and 8.85 with 0.663 sensitivity and 0.629 specificity, respectively. The corresponding AUCs of the TyG index were 0.652 (95% CI, 0.599–0.705; *p* < 0.001) and 0.681 (95% CI, 0.621–0.742; *p* < 0.001) for all-cause and CV mortality, respectively.

## Discussion

In our PD-based cohort study, we assessed the association of TyG index with mortality risk over a mean follow-up period of 3.83 years and observed that a higher TyG index was independently associated with higher risks of all-cause and CV mortality after adjustments of various potential confounders in the Cox models. These consistent associations remained in the sensitivity tests which treated TyG index as a continuous variable or per 1-SD increase. Thus, our findings were valid and robust for TyG index to predict all-cause and CV deaths in PD cohort.

Insulin resistance indicates an impaired biological response to insulin in liver, skeletal muscle or adipose tissues, and its presence in CKD was first demonstrated by Defronzo in 1978 using the gold standard method^20^. IR may even develop in early stage of CKD regardless of its causes and gets worse as renal function declines^21^. The proposed factors of the complex IR in CKD consist of the accompanying comorbid conditions, smoking, sedentary lifestyle, central obesity, uremia milieu, anemia, metabolic acidosis, bone–mineral disease and vitamin D deficiency^22^. Overall, inflammation and oxidative stress seem to be principal determinants in this population. IR is also a well-recognized complication of ESRD patients undergoing hemodialysis or PD through diverse pathophysiological mechanisms. Compared to matched cohorts with normal renal function, dialysis patients had significantly lower insulin sensitivity and IR was strongly associated with the level of triglyceride^23^. Notably, nonalcoholic fatty liver disease (NAFLD), a manifestation of liver IR, was highly prevalent in PD patients and associated with high risk for CV disease^24^.

As a valid proxy of IR, most of previous studies have mainly linked TyG index to the risk of incident type 2 DM. TyG index even appears to be a better predictor than FPG or triglycerides of incident DM in normoglycemic patients^25^. More recently, some investigators have diverted their attention to the association of TyG index with various cardiovascular-related abnormalities, such as hypertension, systemic arterial stiffness, coronary artery calcification, and cardiac autonomic neuropathy and diabetic micro- and macro-angiopathies^26–30^. The TyG index is a predictor for cardiovascular and all-cause mortality in CVD patients with diabetes or pre-diabetes in an American population^31^. Moreover, there is increasing evidence that the TyG index is associated with adverse cardiovascular outcomes in the general population and in many patient populations, such as COVID-19, type 2 DM with acute coronary syndrome, acute ST elevation myocardial infarction^32–34^. A recent meta-analysis of eight cohort studies comprising more than 5 million patients suggested a crucial determinant of TyG index in predicting atherosclerotic cardiovascular disease^35^. Our present findings also suggested a significant relationship between baseline TyG index and subsequent clinical outcomes in patients on PD. In line with previous studies, our study showed that patients in the highest tertiles of TyG index had a 2.78-fold and 2.12-fold higher risk of CV death and overall mortality, respectively, compared with the first tertile. The findings were further strengthened when TyG index was treated as a continuous variable, implying the detrimental effects of IR on patient survival in our PD cohort.

The exact mechanisms underlying the predictive value of TyG index in patient survival have not been well clarified in the present study. Several plausible speculations associated with IR were proposed. First, a high value of TyG index indicates IR in the liver or adipose tissues because FBG mainly reflects IR in the liver, whereas TG mainly reflects IR in adipocytes. IR has been reported to result in endothelial dysfunction, oxidative stress, chronic inflammation and altered coagulation, atherosclerotic plaque formation and concentric cardiac remodeling and left ventricular dysfunction^36,37^. Furthermore, a previous study on assessment of microvascular and macrovascular damage using vascular damage parameters demonstrated a significant association of TyG index with arterial stiffness and microvascular damage^38^. A recent prospective cohort study in a Chinese community showed that TyG index was an independent predictor of the risk of incident CV disease, including stroke and myocardial infarction, which was in part attributed to more severe and complex comorbidities^39^. Taken together, all of the above findings supported our notion that higher TyG index leads to higher risks of CV and all-cause mortality in our PD cohort.

Regarding the optimal cut-off value of TyG index, cohort studies were retrospectively conducted to examine this issue with several clinical outcomes. In a Chinese cohort study, the optimal cut-off value of TyG for NAFLD was 8.5 and the AUC was 0.782 (95% CI 0.773–0.790), with sensitivity and specificity of 72.2% and 70.5%, respectively^40^. Another Korean study showed a cut-off point of 8.718 for TyG index to predict incident metabolic syndrome with AUC of 0.556 (0.531–0.581, *p* < 0.001) and concluded that TyG index outperformed HOMA-IR in predicting metabolic syndrome^41^. In a cross-sectional study of the Chinese population, the cut-off values of the TyG index for prevalent hypertension were 9.04 in men (sensitivity 52.58, specificity 72.27%) and 8.59 in women (sensitivity 76.07, with a specificity of 46.69%)^42^. Recently, an Iranian study reported that the cut-off value of TyG-index for incident CVD was 9.03 (59.2% sensitivity and 63.2% specificity) with AUC of 0.663 (95% CI 0.645–0.681); the value of TyG-index for incident CHD was also 9.03 (60.0% sensitivity and 62.8% specificity) with AUC of 0.669 (95% CI 0.651–0.688), respectively^43^. Thus, the derived TyG cut-off points (8.85 and 8.79) for CV and all-cause mortality among our PD cohort were comparable to those values for detection of CV disease, coronary heart disease and incident metabolic syndrome, NAFLD and prevalent hypertension.

As a retrospective study, there are several limitations to address in the interpretation of our results. First, the causal relationship was difficult to establish for an observational study. Associations are not equal to causations which can only be confirmed in randomized controlled trials. However, we collected various clinical parameters for adjustments, including demographics, pharmacotherapy, comorbidities, biochemical variables as well as PD data. In addition, Cox models were conducted with TyG index across tertiles and as a continuous variable or per 1-SD increase to test the consistency. Therefore, the independent association of TyG index with patient mortality was convincing and consolidated. Second, the study cohort merely included patients in one Taiwanese hospital. Whether our findings can be extrapolated to other ethnic population required further research in multinational and multicenter PD units. Third, due to the lack of fasting insulin levels, the TyG index could be compared with HOMA-IR, which is used to quantify insulin resistance.

In conclusion, our study illustrated that the TyG index was a significant predictor of increased risk of poor clinical outcomes in patients undergoing PD. An increase in baseline TyG index was associated with higher risks of CV and overall mortality independent of a variety of critical confounders. TyG index is an accessible, reliable and cost-effective parameter to assess IR in clinical practice. Based on our findings, the TyG index could be served as an important biomarker to stratify and identify individuals at higher risk of mortality in the hope to improve their survival through lifestyle modification and pharmacotherapy. Whether targeted therapy to alleviate insulin resistance in patients with a high TyG index leads to better survival warrants future research.

## Data Availability

The data sets generated or analyzed during the current study are available from the corresponding author upon reasonable request.
